# Spike Sorting of Muscle Spindle Afferent Nerve Activity Recorded with Thin-Film Intrafascicular Electrodes

**DOI:** 10.1155/2010/836346

**Published:** 2010-03-30

**Authors:** Milan Djilas, Christine Azevedo-Coste, David Guiraud, Ken Yoshida

**Affiliations:** ^1^Vision Institute, 17 rue Moreau, 75012 Paris, France; ^2^LIRMM/INRIA, University of Montpellier 2, 161 Rue Ada, 34095 Montpellier Cedex 5, France; ^3^Biomedical Engineering Department, Indiana University-Purdue University Indianapolis, 723 W. Michigan St - SL220F, Indianapolis, IN 46202, USA; ^4^Center for Sensory-Motor Interaction, Department of Health Science and Technology, Aalborg University, Fredrik Bajersvej 7 D3, DK-9220 Aalborg, Denmark

## Abstract

Afferent muscle spindle activity in response to passive muscle stretch was recorded in vivo using thin-film longitudinal intrafascicular electrodes. A neural spike detection and classification scheme was developed for the purpose of separating activity of primary and secondary muscle spindle afferents. The algorithm is based on the multiscale continuous wavelet transform using complex wavelets. The detection scheme outperforms the commonly used threshold detection, especially with recordings having low signal-to-noise ratio. Results of classification of units indicate that the developed classifier is able to isolate activity having linear relationship with muscle length, which is a step towards online model-based estimation of muscle length that can be used in a closed-loop functional electrical stimulation system with natural sensory feedback.

## 1. Introduction

Functional electrical stimulation (FES) is one solution for restoring movement in paralyzed limbs. In order to correct for disturbances and unavoidable musculoskeletal modeling errors, closed-loop FES is desirable. With the advent of advanced implanted prosthetic interfaces, natural sensors are being explored as an alternative source for feedback information. By directly interfacing the peripheral nerves, it is possible to record signals from natural sensors distributed throughout the body [1–4]. Muscle spindles are one type of natural sensor. Their main function is to signal changes in the length of the muscle within which they reside [5]. Changes in the muscle length are associated with changes in the angles of the joints that the muscles cross. Therefore, their activity can be used to provide feedback information about muscle state in a closed-loop FES system [6], as these sensors remain intact and active below the level of lesion in spinal cord injured patients [7]. In a number of studies muscle spindle afferent activity was recorded using cuff electrodes to provide natural sensory feedback [1, 4, 8–10]. Despite their chronic stability and noise immunity, the use of cuff electrodes is ultimately limited by their low selectivity, which requires them to be distributed throughout the body and in close proximity to the sensory end organs targeted for use in feedback [11]. An alternative to the cuff is the longitudinal intrafascicular electrode (LIFE). It is designed to be implanted longitudinally within the peripheral nerve where it can record activity from a relatively small population of nerve fibers [12–16].

In a previous study a simple corrector controller was implemented to follow a desired joint angle trajectory in the presence of externally applied disturbances [6]. The approach proved to be applicable as feedback in online closed-loop control in restrictive conditions (limited motion speed and range), probably due to the unaccounted variation of dynamic sensitivity of the two different types of muscle spindle sensory endings. Type Ia sensory fibers predominately encode information about the rate of change of muscle length, and type II sensory fibers predominately encode information about the muscle length. The former introduce a component in nerve response that makes the relationship between nerve activity and muscle length velocity dependant. The new generation of LIFE, the thin-film LIFE (tfLIFE) [17], was used in a recent study in which effort was made towards modeling the neural response of muscle afferents to passive muscle stretch [18]. Even though the multichannel tfLIFE provides improved selectivity compared to the older single-channel LIFE, the same effect of velocity dependence in the neural response was observed. Solving this issue would require the decomposition of the multiunit recorded signals into components originating from different fiber types. Isolation of the activity of type II sensory fibers could allow for a linear model approximation to be used to track muscle length variations [19, 20]. In the following we give a brief overview of the state of the art in real-time neural spike sorting of multiunit ENG recorded with LIFE.

Voltage threshold triggering is a common way for detecting neural spikes [21]. This method requires minimal computing power for signal processing and it is also easy to implement. On the other hand it does not always provide acceptable isolation between nerve and noise spikes. A method using the discrete wavelet transform (DWT) for signal denoising was developed by Donoho [22] to detect action potentials buried in noise. It involves thresholding of the detail coefficients in the wavelet decomposition. After denoising, spikes are detected by the simple voltage thresholding method. Eventually, the signals are reconstructed by applying the inverse DWT. The procedure was applied on ENG signals by Diedrich et al. [23] and recently on intraneural signals recorded using thin-film LIFE [24]. In both studies the authors used the Symlet 7 wavelet because of its similarity to typical action potential waveforms in the recordings. No quantitative analysis was given to justify the choice of wavelet and the denoising threshold was chosen empirically. In the latter study cycle spinning was used, a method developed by Coifman and Donoho [25], to reduce but not completely remove the effects of DWT translation variance. Signal detection can also be performed in wavelet space, without the need for reconstructing the signal. That way the required computing power is reduced which is an important issue for real-time implementation. Only being interested in analysis, and not synthesis, also relaxes the constraints on wavelet choice, since not all wavelets are suitable for signal reconstruction. K. H. Kim and S. J. Kim [26] have suggested using multiscale wavelet analysis as an equivalent to multiple approximations of matched filters. Their method utilizes the point-wise product of wavelet transform coefficients over several selected scales. The detection method proposed does not require “quantitative” a priori information on either the target signal or background noise, and only involves qualitative information that is common to the neural signal recordings, that is, spike waveform shapes that are most common. Unlike the discrete wavelet transform, the continuous wavelet transform (CWT) can operate at every scale, from that of the original signal up to some maximum scale that one determines by trading off the need for detailed analysis with available computational horsepower. During computation the analyzing wavelet is shifted smoothly over the full domain of the analyzed function. This eliminates the problem of translation variance when using the DWT. The stationary DWT and its equivalent methods reduce but not completely eliminate this problem [27]. The complex wavelet transform (CoWT) is a complex-valued two-dimensional extension of the standard wavelet transform. It provides a suitable framework to incorporate two wavelets into one transformation, and one wavelet being the real part, and the other the imaginary part of the transform. A recent study demonstrated that spike detection in the auditory nerve based on this approach outperforms a matched filter approach [28].

Detected neural action potentials can be classified using different sets of features. Action potentials waveform shapes depend on neuron type, electrode construction, electrode placement relative to the neuron, and the local properties of the tissue surrounding the electrode. Action potentials from different neurons can be distinctively different, but can also be quite similar. Moreover, high noise levels on weak nerve signals make rapid and accurate classification of spikes challenging. Classification using Fourier transform coefficients as features gives higher classification error rates when compared to methods using time domain features [29, 30]. One method for choosing features automatically is with principal component analysis [31, 32]. The idea behind principal component analysis (PCA) is to find an ordered set of orthogonal basis vectors that capture the directions in the data of largest variation. Real-time implementation of PCA is also possible using transversal filter structures [30]. To the best of our knowledge, there are no results published about the use of PCA on intrafascicular ENG recordings. McNaughton and Horch reported that classification using artificial neural networks (ANNs) outperformed both methods using time-domain features and methods using Fourier transform coefficients [33]. In another study the ANN approach managed to classify 6 out of a total of 10 units present in the signals. With more than 10 units present, the number of separable units dropped [34]. In these studies a three-layer feedforward ANN was used. The main drawback of using ANNs is that the results of classification are very sensitive to the data set used to train these networks, and obtaining a “good” training set is difficult, especially in the case with low SNR [26]. Moreover, there are no criteria for defining the appropriate structure and size of an ANN. Many trials are necessary to obtain good results. At the end, it is uncertain whether acceptable results are obtained or not. Moreover, amplitude and shape of neural spikes change over time, due to electrode drift and fibrous encapsulation of the electrode recording sites (tissue reaction to implantation). Classifiers based on artificial neural networks would require tedious and supervised relearning procedures which are conducted in laboratory conditions [35]. This would not be very practical in real-life working conditions of neuroprosthetic devices.

Independent component analysis is a method that can improve source separation by taking advantage of multiple channels available. However, one restrictive assumption of this approach is that the minimum number of channels must equal the number of sources [21]. It will be shown later that the number of units picked up by one channel of the LIFE is greater than the number of available recording sites on the electrode. Methods for source separation based on the relative difference between spike features recorded by different channels [36] are not applicable in the case of the LIFE either. Analysis of recorded data shows that the different channels need not pick up activity from the same units.

In this paper we present a novel approach to neural spike detection and classification based on the continuous wavelet transform using complex wavelets.

## 2. Methods

### 2.1. Acute Animal Experiments

Acute rabbit experiments were conducted on 10 New Zealand white rabbits (median weight 4 kg and standard deviation 0.24 kg) to acquire experimental data for validating the spike sorting algorithm.

#### 2.1.1. Animal Preparation

Anesthesia was induced and maintained throughout the experiments with periodic intramuscular doses of a cocktail of 0.15 mg/kg Midazolam (Dormicum, Alpharma A/S), 0.03 mg/kg Fetanyl and 1 mg/kg Fluranison (combined in Hypnorm, Janssen Pharmaceutica). In order to immobilize the left leg of the rabbit, it was anchored at knee and ankle joints to a fixed mechanical frame using bone pins placed through the distal epiphyses of the femur and tibia. The common calcaneal tendon was attached to the arm of a motorized lever system (Dual-mode system 310B Aurora Scientific Inc.) using a yarn of polyaramid fibers (Kevlar 49, Goodfellow Cambridge Ltd). The motorized lever system provided both the actuation and measuring. Pulling the Kevlar fibers produced ankle extension and releasing tension on the Kevlar fibers resulted in ankle flexion (stretched muscle returning to its resting state due to its intrinsic elasticity). Fixation to the mechanical frame insured the elimination of mechanical vibration that might have resulted from a free swinging foot. A tripolar cuff electrode was implanted around the sciatic nerve. It was used to find the length-tension curve for the medial gastrocnemius (MG) muscle. Electrical charge was delivered using a stimulation unit (Grass Technologies SD9), coupled with a photoelectric isolation unit (Grass Technologies PSIU6). The nerve was stimulated with 300 microseconds pulses and a pulse repetition frequency of 2 Hz. The stimulation intensity was set to the level that produced maximal nerve twitch response (maximal amplitude of compound action potential). Keeping the stimulation level constant, muscle length was varied in small incremental steps. Isometric force produced by the stimulated muscle was simultaneously monitored. 

A tfLIFE structure was implanted in the tibial branch of the sciatic nerve innervating the MG muscle of the rabbit's left hind limb. It was located 3 cm distal to the cuff electrode implantation site. The tfLIFE enabled the monitoring of multichannel ENG from the fascicle in which the structure was implanted. By having the electrode implanted very close to the muscle, chances of having anything except muscle spindle activity recorded are minimized. Moreover, in order to have purely muscle afferent activity in the recordings, the sciatic nerve was crushed proximally of the cuff and tfLIFE implantation sites using a pair of forceps. It should also be mentioned that with increasing levels of anesthesia, the effects of reflex mechanisms diminish, and decreased tonic stiffness of muscles is observed [37, 38]. As for Golgi tendon organ activity that encodes muscle force information, it accounts for only a small part of the muscle afferent signal under passive conditions. A histological study reports that they account for less than 5% of the total number of receptors in tibialis anterior muscle [39]. 

Animals were euthanized at the end of the experiments. All procedures used in experiments were approved by the Danish Committee for the Ethical use of Animals in Research.

#### 2.1.2. Data Acquisition System

The amplification system consisted of a low-noise preamplifier (AI402, Axon Instruments), followed by a gain-filter amplifier (Cyberamp 380, Axon Instruments). Signals were recorded using a custom modified multichannel digital tape recorder (ADAT-XT, Alesis). ENG data were band pass filtered (4th order Bessel, corners at 0.1 Hz and 10 kHz), amplified (gain 5000), and acquired with a sampling rate of 48 kHz per channel. Out of the eight available electrode sites on the tfLIFE, only the four having the lowest background noise level were selected to be recorded from, due to the limited number of available channels on our recording system. 

Signals for driving the motorized lever system (Aurora Scientific) were generated on a portable computer using LabVIEW (National Instruments). Before feeding the analog signal into the lever system input, the signal passed through a low-pass filtering stage (corner at 100 Hz) in order to remove any quantization noise resulting from the D/A conversion. Quantization noise would translate into vibration of the lever arm which could have induced activity of muscle spindles [40, 41]. Length and force signals were recorded simultaneously with the ENG. 

#### 2.1.3. Muscle Stretch Protocol

The recorded nerve activity is a mixture of activity from two sensory fiber types. A convenient and common way for studying muscle spindle afferent response is applying sinusoidal extensions to a muscle and simultaneously recording the muscle receptor afferent ENG [40, 41]. With recordings made with such a protocol, it is later possible to analyze the contributions of the two components to the aggregate recorded activity. In our experiments the MG muscle was passively stretched by rotating the ankle in the extension/flexion plane using the lever arm. The initial muscle length was set to the muscle length at which the produced isometric force was maximal. Ankle position was set so it was flexed 90° and then finely adjusted by experimentally finding the maximum of the length/tension curve.

The muscle was presented with sinusoidal stretches of 2 frequencies: 0.01 Hz and 0.25 Hz. Both stretch profiles had peak-to-peak amplitudes of 4 mm which covered a large portion of the normal range of motion of the ankle. The two frequencies were selected to model slow movements, such as slow walking (0.25 Hz) and correction for postural control (0.01 Hz). They were selected to be below a critical frequency corresponding to a system pole related to the activation and conduction delays of the neuromuscular system at 1-2 Hz. The maximum frequency in the pass band of the system is about 1/5 the pole frequency [6]. The faster sine frequency was chosen accordingly. According to Kralj and Bajd, FES assisted walking with minimal energy cost is between 0.35 and 0.56 m/s [42]. If we use FES gait with stride length of 1 m, then the frequency is between 0.35 and 0.56 Hz. Regarding body sway, it has been reported that most of the energy in able-bodied body sway is in the band between 0.02 and 0.2 Hz [43, 44]. It is more difficult to estimate low frequencies because of the lack of velocity sensitivity and lower levels of neural activity. We chose lower frequencies to demonstrate a worst case scenario. The durations of the recordings were 2 minutes for the slower stretch (to allow for one full cycle of the sinusoid to complete), and just over 1 minute for the faster stretch (4 cycles of the sinusoid). Simultaneous recordings were made from the four intrafascicular electrode sites, together with force and position recordings from the muscle lever system. The muscle was not stimulated while being stretched.

### 2.2. Neural Spike Detection

The methodology for spike detection we developed is an expansion of the idea of using complex wavelets, so it covers a range of temporal scales. Contrary to the matched filtering approach, where a priori information is necessary about action potential shapes, using the multiscale complex wavelet approach provides multiple approximations of matched filters, making it a more generic approach and probably more robust when addressing the issue of changes in the spike waveforms. A training set of action potentials waveforms is therefore not necessary, which is an advantage of the wavelets approach over using artificial neural networks. 

#### 2.2.1. Choice of Complex Wavelet Family and Scale Factors

The following equation defines the wavelet transform *W*:

(1) $$W\left(\alpha ,\tau \right)=\int_{-\infty }^{+\infty }x\left(t\right)\frac{1}{\sqrt{\alpha }}\Psi \left(\frac{t-\tau }{\alpha }\right)dt,$$

where the real numbers **α** and **τ** denote scale and translation, respectively. The wavelet transform essentially performs a correlation analysis between the input signal *x* and the translated and dilated version of a reference signal called the mother wavelet Ψ. Hence, it would be expected that the output would have local maxima where the input signal most closely resembles the analysis template, that is, the wavelet function. Some wavelet basis functions are similar in shape to neural action potentials. In addition, the basis function is dilated over a range of scales. If the scales are well chosen, the wavelet transform can act as a number of effective approximations of the matched filter, even though the exact action potential waveforms are not known. In the case of complex wavelets the mother wavelet function *Ψ* is complex and the wavelet transform is also complex. 

**Figure 1:**
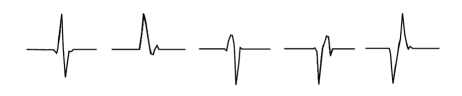
Five distinctive action potential waveforms extracted from experimentally recorded data. Length of each trace is 1 milliseconds.

**Figure 2:**
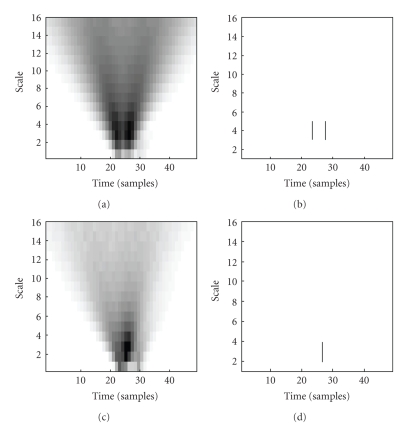
Time-scale plots of two action potential waveforms with different waveform shapes (a, c) and the same plots showing only wavelet coefficients larger than 95% of the coefficient with the maximal magnitude (b, d).

In order to find the optimal complex wavelet, around 30 action potentials with different waveforms were visually identified by inspecting the recorded ENG data and extracted. Five action potentials with distinctly different shapes are shown on Figure 1. The CoWT was computed for all extracted waveforms using a series of complex wavelet families available in the MATLAB wavelets toolbox (The Mathworks). The computations were done using a range of scales to find the optimal scales, which produced the wavelet coefficient with the maximal magnitude. Optimal scales were selected to be the ones that produced at least one coefficient with a magnitude larger than 95% of the maximal CoWT response among all scales. An example of time-scale representations of two action potentials with different shapes is shown on Figure 2. In this example, the CoWT was computed using the cgau1 wavelet using scales from 1 to 16. Panel (a) shows coefficient magnitudes for the first action potential waveform. Panel (b) shows only the coefficients above the 95% threshold. On panels (c) and (d) are the corresponding plots for the other action potential waveform. The complex Gaussian family and the complex Morlet family of wavelets both produced well localized peaks in CoWT space, that is, these peaks appeared with a small or zero delay relative to the position of the waveform peaks in the time domain. In terms of the magnitude of the output coefficients, the complex Morlet family produced wavelet coefficients with a 50% lower magnitude compared to the complex Gaussian family. This was consistent for all extracted action potentials. With the complex Shannon family both the peak localization and the magnitude of the output coefficients were poor. Therefore, only the complex Gaussian family of wavelets was considered in the process of selecting the optimal wavelet to be used for spike detection. 

**Figure 3:**
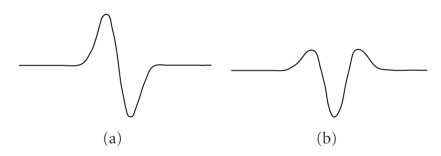
Real (a) and imaginary (b) parts of the cgau1 wavelet.

Within the complex Gaussian family, as a general rule, higher-order wavelets produced wavelet coefficients with lower magnitudes, for example, cgau1 produced a larger response than cgau2, and cgau2 produced a larger response than cgau3 and so on. Consequently, the cgau1 wavelet was chosen as optimal for neural spike detection. The real and imaginary parts of the cgau1 wavelet are shown on Figure 3. The optimal scales range was from 1 to 6. 

For comparison, the above analysis was also performed using non-complex wavelets that support CWT, from which the db2 wavelet produced wavelet coefficients with maximal magnitudes. Compared to the cgau1 wavelet, the range of scales for the db2 wavelet was larger more than two times (scales from 2 to 16). Comparing the inter-quartile ranges of the scale factors, the cgau1 wavelet requires 3 times less scale factors in order to cover all action potential waveforms. This is important when later looking into the implementation of the algorithm. If the number of scale factors were the same, the cgau1 wavelet would require double processing time compared to using the db2 wavelet. As the number of scale factors for the db2 wavelet is more than double, implementing the algorithm using the cgau1 wavelet would require less processing time. 

Another benefit of having a smaller range for scale factors is the fact that it should result in better detector specificity. Using the analogy that low scale factors correspond to low frequencies in the signal spectrum and higher scale factors to higher frequency components in the signal spectrum, then a wider range of scale factors would correspond to a wider frequency bandwidth of the transforms and thus there would be more noise influence on detection performance. We will show this to be correct later on. 

#### 2.2.2. Algorithm Implementation

Prior to detection, ENG signals were band pass filtered to remove noise and artifacts. Implementing a matched filter to remove powerline noise in real time is also a possibility. The algorithm needs to be robust enough to adapt to the changing parameters of the powerline noise. Not only does the amplitude of the harmonics change, due to multipath propagation, but so does the frequency [45]. Fitting a sine wave of 50 Hz and a number of its harmonics on the raw data and subtracting the fit give good results. Fitting and subtraction of noise harmonics up until the 5th or 6th harmonic of the noise was sufficient to remove slow baseline oscillations. Performing the fit on 20 millisecond windows (corresponding to 50 Hz) insureed that the fitting algorithm locks onto the phase of the noise. This value is also suitable for real-time implementation since it is below the delay that can be tolerated in closed-loop FES control.

The detection algorithm consisted of finding peaks in the signal transformed into wavelet space that crossed a preset threshold. Wavelet coefficients were computed only at optimal scales. In order to have the scales independent of length of the signal being processed, a windowed continuous wavelet transform was implemented. The transform was computed using a 20 millisecond moving window, matching the window duration from the noise removal step.

Depending on the shape of an action potential, there were cases where computation of the wavelet transform resulted in maxima from different scales to appear at different time instants—if more than one wavelet coefficient had a magnitude larger that a preset detection threshold, multiple detections of the same neural spike occurred. The effect of multiple peaks in wavelet space is illustrated on Figure 2, where for one action potential the maxima are localized at a single time instant (one vertical bar on the panel (b)), while for the other waveform the maxima appear in 2 time instants (2 vertical bars on panel (d)). In the latter case there would be two events detected above the threshold for only one action potential. In order to avoid this, a refractory period was introduced into the algorithm: when a spike is detected, another event can be registered only after the expiration of the preset time interval. (Not to be confused with the nerve fiber refractory period. This refractory period, implemented in the algorithm, is a period during which any other peaks crossing the detector threshold are ignored). Exploratory data analysis on all extracted action potentials showed that the scattering of wavelet coefficient maxima was never larger than 146 microseconds. The refractory period was therefore set to this value.

### 2.3. Classification of Action Potentials

It is important to note that our objective was not to classify all the action potentials to eventually have accurate information about single-unit activity, but rather to isolate activity from subsets of fibers that would be usable for closed-loop FES. In other words, we were interested in classes that would provide a linear relationship between neural firing and muscle length and therefore independent of muscle stretch velocity. 

In principle, there are two approaches when wavelets are used in pattern recognition problems. The first is to use one wavelet to represent all spike shape variations, and the other is to use different wavelets for each of the spike templates. In the former case one wavelet may not be sufficient if there is a large variation between the action potentials waveforms. This is very probable if a larger number of units are present in the recording. In the latter case, the best representation of different spike waveforms would be achieved by designing new wavelets for continuous wavelet transform. The procedure consists of approximating a given pattern using least squares optimization under constraints leading to an admissible wavelet [27]. We have tried this on spike waveforms extracted from experimentally recorded ENG, and good fits could not be found for all waveforms because of the imposed constraints. Only certain biphasic action potentials produced good fits. Even if a set of wavelets could be found that represents all spike waveforms, the computational power required for real-time parallel computation of CoWT coefficients and processing would exceed the computing power available today. A compromise between the two approaches could be the representation of the full set of action potential waveforms by a reduced set of wavelets, for example, using complex wavelets. Two different action potential waveforms would be represented by the real and imaginary parts of a complex wavelet. 

The multiscale CoWT has an advantage that it also offers a framework for classifying the detected neural spikes. Action potentials differ in their shape and amplitude and it was necessary to choose a feature set and a distance metric with which they would be distinguishable. Exploratory data analysis on the extracted neural spike waveforms indicated that the CoWT coefficients computed using the same range of scale factors as in the detection algorithm could be suitable as classification features. Visual inspection of time-scale plots, like those shown on Figure 2, indicate that they are different for different spike waveforms. Classification could therefore be performed by creating feature vectors using the computed wavelet coefficients and then clustering the data using the Euclidean distance metric. Feature vectors were created by concatenating rows of the time-scale plots for the real and imaginary parts of the CoWT transform, where each row consisted of the CoWT coefficients from a particular scale. 

In our context, there are two steps in the classification of detected action potentials. The first is the calibration phase, where the classification is performed offline. The purpose of the calibration is to identify action potentials that encode relevant information for closed-loop control. In other words, we look for classes of action potentials having a linear relationship between firing rate and muscle length. Once they are identified, the second step of the classification is to recognize and track these particular action potentials online in order to estimate muscle state. 

Action potentials were classified using k-means. In order to avoid local minima in the optimization, clustering was repeated 50 times (replicates), each time using different starting points. Clustering using less replicates sometimes produced different clustering results using the same data set. After the classification was complete, firing rates of each class were computed. Eventually, the linearity of the relationship between firing rates and muscle length was checked for each class. 

One requirement for using k-means algorithm is to know in advance the total number of classes. In order to estimate the number of units each site of the tfLIFE picks up activity from, aggregate afferent firing rates of the postprocessed ENG signals were computed by counting the number of peaks above threshold in a 1-ms moving time window. This window duration matches approximately the absolute refractory period of mammalian sensory nerve fibers [46]. Since there cannot be two spikes originating from the same axon within this period, an estimation of the minimal number of axons can be made by counting the number of spikes in the window. Miscounts can occur in cases where two or more spikes overlap. By extending the above analysis onto a number of periods of the faster sine wave, the probability of spike overlap becomes smaller, as it is unlikely that the same subset of spikes will overlap in each period at the same phase. The number of units being picked up by the recording electrode changes depending on how much the muscle is stretched. The more the muscle is stretched, the more units are firing. Values in the Table 1 are estimates of the number of units at the time the muscle is maximally stretched. No significant differences in the numbers are found between the slower and faster sine wave data. On the other hand, there is a large variation when comparing between rabbits and in some cases between different channels of one electrode. Statistically, the median number of units picked up at maximal stretch is 8, with a standard deviation of 2.7. To account for the changing number of classes, the total number of classes for off-line k-means clustering was set to 10, allowing the algorithm to create empty classes.

### 2.4. Evaluation

**Figure 4:**
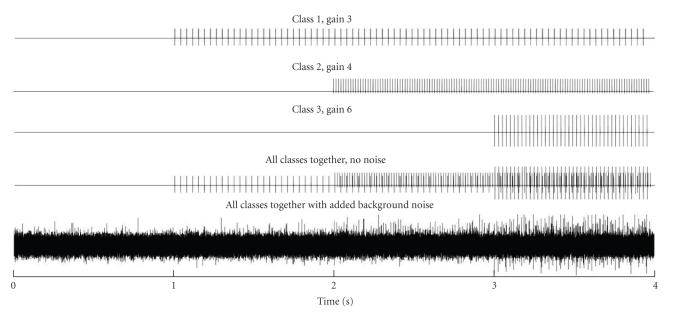
Steps in the creation of a synthetic signal. Top 3 traces are the generated action potential trains for 3 units. The onset of the first spike train is at *t* = 1 second, the second train at *t* = 2 second, and so on. The fourth trace is the superposition of traces 1–3. Background noise is eventually added resulting in a signal on the bottom trace.

In order to be able to evaluate the performance of the spike sorting algorithm, knowledge of the exact timing and class of each action potential in the ENG signal is needed. Because of uncertainty about this information in experimental data, artificial signals based upon recorded action potentials were synthesized. Five action potentials with distinctly different shapes were chosen to represent 5 neural spikes originating from different axons, that is, different spike classes. These are shown on Figure 1. The waveforms were normalized and used to synthesize spike trains. Spike train firing rates were randomly chosen from within the range found in the literature [47]. With the exception of burst firing, muscle spindle afferents can fire with a rate up to about 75 Hz. Burst firing was not considered because the amplitude of an action potential firing in burst mode can vary often as much as 50% [36]. Spike amplitudes were scaled by integer values ranging from 3 to 6 standard deviations of the background noise level, which corresponds to the range of values found by inspecting the recorded data. These 4 scaling factors represented different SNR levels for which the analysis was performed. Therefore, the SNR is defined here as the ratio of the peak amplitude of the noise-free action potential and the standard deviation of background noise. Signals were synthesized by adding the spike trains onto experimentally recorded background noise. Signals with up to 10 units firing simultaneously were synthesized. Spikes having the same waveform and amplitude were considered to be from the same axon (belonging to the same class). A total of 900 signals were generated: 100 signals with 2 units active, another 100 with 3 units, and so on until 100 signals with 10 units. Steps in the creation of one synthetic signal with up to 3 units active are shown on Figure 4.

## 3. Results

**Figure 5:**
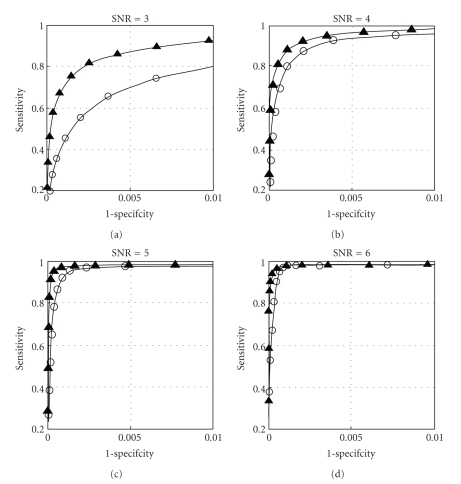
ROC curves for four SNR levels. Performances of a simple threshold detector (empty-circle line) and the wavelet-based detector (full-triangle line) are compared.

Performances of the wavelet-based detector and the detector using simple amplitude thresholding are shown on Figure 5 in the form of receiver operating characteristics, or ROC curves. These curves are graphical representations of detector sensitivity versus specificity using a range of detection thresholds. On the whole range of SNR levels the wavelet-based detector outperforms the detector based on amplitude thresholding; that is, for any given specificity, the corresponding sensitivity is greater for the wavelet-based detector. The performance gap becomes especially prominent with low SNR. 

**Figure 6:**
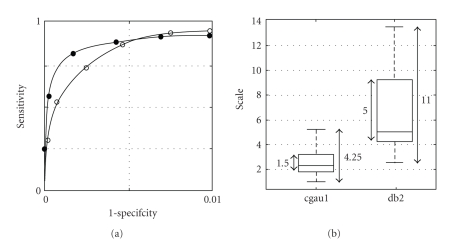
(a) ROC curves for the detector based on the non-complex db2 wavelet (open-circle curve) and the detector based on the cgau1 wavelet (full-circle curve). (b) Ranges of detection scale factors for the cgau1 and db2 wavelets. The increment between successive scales is 0.25.

Compared to detection using the non-complex db2 wavelet, detection using the cgau1 wavelet shows better specificity in ROC space (Figure 6(a)). This is most probably due to the wider range of wavelet transform scale factors required for the db2 wavelet (Figure 6(b)). A wider scales range translates into a wider frequency bandwidth, as explained earlier. For the cgau1 wavelet the scales range was from 1 to 6, and for the db2 wavelet it was from 2 to 14. 

**Figure 7:**
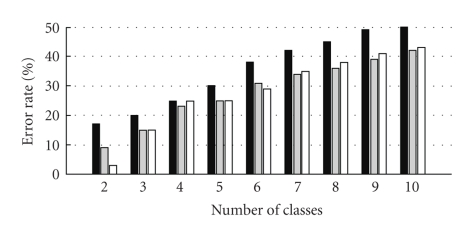
Classification error rates depending on the number of units simultaneously active, compared for three classification approaches: template matching (black), principal components analysis (gray), and wavelet-based (white).

Classification results are shown in the form of classification error rates which are ratios of the number of misclassified spikes to the total number of spikes being classified. The wavelet-based classification is compared to two other methods of classification: principal components analysis (PCA) and template matching. Results are shown on Figure 7 starting from the case when only two different spike classes are present in the signal up to the case where 10 units are simultaneously firing. Classification based on template matching produced the highest classification error rates, while wavelet-based and PCA-based approaches showed similar results. 

The spike sorting technique was eventually applied on experimentally recorded muscle spindle afferent nerve activity. Only flexion periods of ankle joint motion (stretch periods of the MG muscle) were analyzed. The detection threshold was chosen to be seven times the standard deviations of the background noise level (in wavelet space). Throughout all the trials, this threshold value corresponded to the point on the ROC curves, where the specificity starts to rapidly deteriorate while at the same time there is little improvement in sensitivity. 

**Figure 8:**
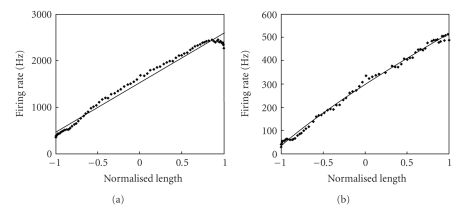
Afferent neural firing rate versus muscle length. Aggregate activity of all detected spikes (a) and activity from 2 clusters having a good linear fit to the data (b) were used to compute the firing rates. Linear regression analyses are the full lines. Muscle length was normalized by 4 mm.

The detected units were classified into 10 clusters. The analysis was performed on data from all rabbits. Two to three spike classes per rabbit showed a linear relationship between their computed neural firing rate and instantaneous muscle length. Since this relationship was not linear when using the aggregate afferent firing rate, the result is an indication that the algorithm is capable of isolating activity of units less sensitive to muscle stretch velocity. Results from one rabbit are shown on Figure 8. (a) shows the aggregate firing rate of all detected spikes. The relationship is clearly not linear in the region where the muscle stretch velocity slows down rapidly (region where normalized muscle length is close to 1). (b) shows the same relationship, but this time using only the activity of the fibers insensitive to the velocity of muscle stretch. A linear regression analysis performed on both shows that the fit on (b) is better. 

## 4. Discussion

Results show that the CoWT is the preferred method for neural spike detection. Even though this wavelet-based classification does not show improvement in error rates compared to the PCA-based algorithm, the advantage of using the wavelet-based approach is that it provides a unique framework for both spike detection and classification, that is, after computing the complex wavelet coefficients in the detection stage, no additional computation is required in the subsequent classification. 

### 4.1. Cluster Centroid Comparison for Different Stretch Rates

**Figure 9:**
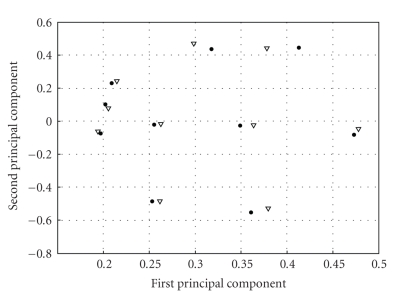
Comparison of cluster centroids for the slow (full circles) and fast (triangles) muscle stretch rates.

It was of interest to compare activities of the same units for the slow and fast muscle stretch rates in order to see if there is a change in their velocity sensitivity. However, it is difficult to identify same units in the 2 stretching conditions. Even if units have a similar shape, there is no guarantee that they are from the same sensory neuron. As we have seen from the extracted waveforms, there are fewer distinctive shapes than there are action potentials in a recording. Nevertheless, comparison of cluster centroids from the 2 data sets was done. In order to graphically present cluster inter-distance, PCA was used to reduce the dimensionality of the time-scale signatures of cluster centroids. Results for one rabbit are shown on Figure 9. Full-circle and empty-triangle markers correspond to cluster centroids from the slower and faster sinusoidal muscle stretch, respectively. Pairs of centroids are easily identifiable, which is a strong indication that they correspond to the same sensory neuron. When relationships between firing rate and muscle length are plotted for the cluster pairs, firing rates from the slower muscle motion exhibit linear or close to linear relationships with length, while firing rates for the faster muscle motion show obvious nonlinear behavior. Under the assumption that the cluster pairs correspond to the same sensory neurons, one could conclude that classes showing different behavior at different muscle stretch rates originate from group Ia sensory fibers, while classes showing linear behavior regardless of the stretch rate originate from group II sensory fibers. There is however not enough evidence to conclude that the units that keep their linear behavior during the faster stretch rate are velocity insensitive. Even the 0.25 Hz muscle stretching is a very slow rate. If a definite conclusion is to be made, the behavior of these units needs to be studied using faster stretch rates than those used in this study.

### 4.2. Aggregate Compared to Single-Cluster Activity

**Figure 10:**
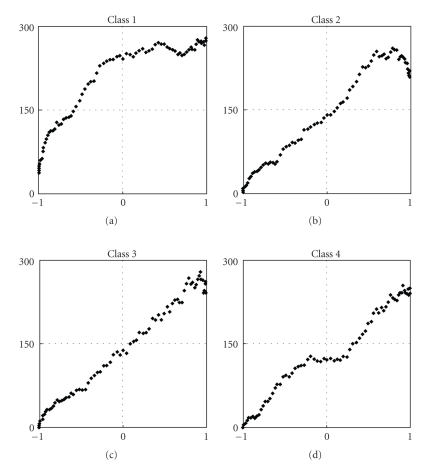
Typical profiles of firing rates versus muscle length for single clusters. On each plot the abscissa is the normalized muscle length and the ordinate is the firing rate in spikes per second.

In cases where the relationship between the aggregate firings rate and muscle length from the faster sinusoidal stretch is nonlinear, activity of single classes exhibits different behavior. A few distinctive cases are shown on Figure 10. Class 1 shows the typical behavior of a sensory neuron with a low activation threshold and saturation point before maximal muscle extension is reached. Activity is registered even at minimal muscle stretch and saturation occurs even before the half of the muscle stretch range. The second class illustrates the difference between saturation and velocity sensitivity. Instead of maintaining a constant firing rate after saturation, with further increase in muscle length the rate starts dropping at maximal stretch, where the velocity of the sinusoidal movement decreases. The third plot is an example of a class with a linear firing rate throughout the whole range of motion. These are the action potentials we are interested in. The fourth plot most probably represents 2 action potentials with similar waveforms classified in one single class. The first starts firing as soon as the muscle starts stretching, after which it quickly saturates. As the muscle is stretched further, the second starts firing and continues firing until maximal muscle extension is reached. Classes with this kind of behavior could also be useful for control purposes as long as their activity can be modeled as a piece-wise linear function. 

### 4.3. Application in Closed-Loop FES

Results show that the spike sorting algorithm may be useful in closed-loop FES using natural sensory feedback. The spike sorting scheme seems to be capable of isolating the activity of secondary sensory endings from the aggregate neural activity of muscle spindle afferents, making it possible to establish a linear relationship between muscle length and neural firing rate. This result is a step towards an online model-based estimator of muscle length. The more classes having linear behavior are found, the more robust the estimation of muscle state would eventually be. Information about cluster centroids from the calibration step would be used as initial values for the classifier and each detected spike would be assigned to one of these initial clusters. Cluster centroid for that class would be updated, taking into account the signature of the new class member. Updating insures the algorithm can adapt to any slow changes in the shape of action potentials, resulting from electrode drift or fibrous encapsulation of the electrode recording sites. Lastly, not all classes would be needed. Only classes relevant for feedback purposes would be tracked. 

To give an idea of the computational requirements, off-line computation of complex wavelet coefficients at 16 scales using the cgau1 wavelet took approximately 4 seconds for 100 ms of data (update time in closed-loop control). It is expected that the processing time would be reduced approximately tenfold when the algorithm is implemented using a low-level programming language, and even more if implemented using an application-specific integrated circuit (ASIC). Therefore, it is expected that real-time implementation is possible. 

Regardless of the spike algorithm, the overall performance of spike sorting is highly dependant on the quality of signal recording. Apart from signal processing methods to minimize the effects of noise, work on novel signal acquisition techniques [48] would be beneficial.

## 5. Conclusions

The CoWT offers a convenient framework for neural spike detection and classification: (1) it is time invariant, unlike the undecimated DWT, (2) it needs 3 times less scale factors compared to the CWT to achieve the same performance in terms of spike detection and localization, (3) it requires less computing power (50%), and (4) it is covers a narrower frequency band, resulting in processing that is less sensitive to noise. Contrary to the previous work [24, 26], no inverse wavelet transform and no additional computation is necessary in the classification stage as we propose a classification scheme based on the wavelet coefficients used to detect spikes. By doing so, computation cost is reduced, and detection and classification are unified in a single data processing flow.

Evaluation on synthesized multiunit ENG shows the wavelet-based neural spike detection outperforms the threshold detection method, especially in cases of low SNR. Results are even better when comparing the CoWT to CWT.

We demonstrated that the complex wavelet-based classification is able to isolate afferent muscle spindle activity having a linear relationship with muscle length. Results are consistent in all experiments. This is a step towards an online model-based estimator of muscle length that can be used in a closed-loop FES system with natural sensory feedback. We show that by using a single signal processing method based on the complex wavelet transform, it is possible to estimate the length of a muscle based on ENG recordings.

The algorithm could be further optimized in terms of computational speed. Reducing the number of scaling factors used to compute the CoWT could perhaps produce negligible deterioration in performance. We were conservative when making the initial choice in order to demonstrate feasibility and to focus on the method itself. This potential improvement will be investigated in more detail before proceeding with the real-time hardware implementation of the spike sorting scheme. 

## Figures and Tables

**Table 1:** Estimates of the number of units.

	0.01 Hz sinusoidal stretch				0.25 Hz sinusoidal stretch			
Rabbit	Ch 1	Ch 2	Ch 3	Ch 4	Ch 1	Ch 2	Ch 3	Ch 4
1	10	9	10	8	9	9	9	6
2	13	13	10	12	12	12	9	12
3	7	8	5	6	7	7	6	7
4	7	8	5	6	7	7	4	6
5	15	15	5	3	14	14	4	3
6	8	9	8	8	7	7	8	8
7	6	4	7	10	6	3	6	8
8	5	11	8	12	6	10	10	11
9	11	9	8	8	10	9	8	8
10	9	7	7	9	8	8	7	9

Estimates of the number of units from which the electrode picks up activity at the point when the muscle is maximally stretched. Results are shown for each channel for both sinusoidal frequencies, for all 10 experiments.
